# Somnambulism and Clairvoyance

**Published:** 1857-07

**Authors:** 


### *Somnambulism and Clairvoyance.*

[Abstract of an Address delivered before the New London County (Ct.) Medical Society, April 9th, 1857, by Joseph Comstock, M. D., and communicated to the Boston Medical and Surgical Journal by vote of the Society.]

Gentlemen—In reflecting upon what subject to address you, the words occurred to my mind, *tell what you know yourself, instead of telling what others know.*

I began the practice of my profession in Rhode Island, in copartnership with Dr. Joshua Perry. Dr. P. was called to a girl aged about 15, then on Conanicut Island, near Newport, but whose parents resided in North Kingston. This girl stated that she had been bitten by a spider, and had strange fits, in which she inclined to strike her thorax with the ends of her fingers, and with such violence that her attendants placed their hands on her breast to prevent her hurting herself. But here commences a peculiarity of her case quite remarkable; it was this, that by touching the hands of persons thus placed, when her eyes were closely shut, she could distinguish one person's hand from another's and that she preferred the hands of her father to those of other persons, and would push a stranger's hands away, and feel for his.

This statement was made to me by Dr. Perry, before I saw her myself; and when I did see her, I witnessed phenomena still more rare and wonderful. It seemed as if the sight of her eyes was transferred to the ends of her fingers, and this faculty was most impressively exemplified; for she could, and did, in my presence, by the touch and with her eyes closely shut, describe colors as precisely as any one could do with their open eyes, and even of complex mixtures. But to make the matter entirely clear, I had a pillow held closely before her face, without in the least impairing her faculty of describing colors. More exquisite still; by feeling the hands of different persons, and those persons afterward handing her a piece of money, she would, and did repeatedly, tell from whose hand it came. The propensity of preferring the hands of her father and other relatives, lasted for several weeks when, like the changes, aversions, antipathies and unaccountable contrarieties of crazy and hysterical patients, it suddenly changed, and even the touch of any one of her kindred would increase her cruel spasms to a horrible height.

As some Italian writer (Baglivi, I believe) had recommended music for the fits, cramps and spasms of persons bitten by spiders, it was procured for Nancy, and when I saw her a second time it was made on a violin by an excellent performer. Music did not cure her convulsions, but it reduced them to order, and to a regular *dancing* system. She danced with all her might and main; spasmodically, laboriously, but involuntarily, and would thus continue the exercise till she was quite exhausted, unable to stand, and would fall to the floor unless caught and supported by some one. If the tune changed, so did her step.

The Italian physicians tell of curing *tarantismus* by music and dancing; but in the case of Miss Hazard it did not cure, but greatly alleviated, and after a month or more of suffering she finally recovered in as strange a manner as she had been afflicted. This was by the formation of an ulcer on the back of her hand, where she said the spider had bitten her, without any *cul de sac*, or abscess. The matter discharged was the greatest novelty; it was thick and green, and without any purulent, bloody, or serous hue, and exactly resembled the juice of green-corn leaves and sage, which dairy women used in making sage-cheese.

An account of this strange case I gave in a letter to one of my medical friends, who sent it to the editors of the *American Medical Repository* (in the year 1803), and in that first of all the American medical periodicals it was published, Hexade the second, Vol. first, Article first, with more minute particulars than I have here entered into. It was from that work, as I have been informed, re-published in Europe; and by some was thought the strangest case on record.

But since then, the cases of Rachel Baker, Jane C. Rider, and Mrs. Cass (the two latter of which were published in the Boston Medical and Surgical Journal), exceed, perhaps, mine in point of marvellousness. I have supposed that this faculty of telling colors by the touch must be imputed to a transfer of the power of vision, or, if you please, a *metastasis*, from the eye to the fingers. In the case of Mrs. Cass, this power seems to have been transmitted to every part of the head; insomuch that no movement could be made in the room, even to the moving of a vial, if her back was toward it, but what she was sensible of and would notice and describe.

Bats are said to have this perception, which has been called the *sixth sense*. Dr. Pardon Bowen, of Providence, many years since informed me that the experiment had been tried, of digging the eyes of a bat entirely out, and then letting it fly in a room where cords and lines were made to cross each other in every possible direction, and that it would fly clear of touching or coming in contact with a single one, or even hitting the walls or top of the room.

The transfer of the senses is not an affair entirely new, but being so extremly rare has not always been credited.

Dr. Rush, who seldom omitted to notice any fact, however strange, which threw a ray of light upon his profession, has noticed the case of a woman who lived near Lyons, who had a confusion of all the senses. She *tasted* with her *touch*, and *heard* with her *eyes* when her ears were closely stopped!

At one of my visits to Miss Hazard, I found her in rather a severe fit, lying on the bed, and witnessed her extraordinary tact, or diseased irritability, with respect to her blood relations, who had all become utterly obnoxious to her touch. At this time her intelligent musician thought, from her moving her feet, that she ought to be helped up to dance. At that instant one of her distant relations came in to see her; she was blind and speechless, and could not have known by her natural senses, that any one had entered. This relative hearing what was said about her being helped up, attempted to assist her, but the effect of his touch was an instantaneous increase of her spasms and convulsions, so that I for a moment was apprehensive of her immediate death. He was at once peremptorily desired to desist. Not being her relation myself, I helped her on to her feet, the music struck up, and I stood by her for about an hour and a half, during which she continued dancing with such violent and exhausting energy, that she would have fallen several times had I not supported her; but after resting a minute, not more, she would resume, or rather her modified convulsions would resume, the exercise with all vigor and energy. As none of her relations could now assist her in her fits, her family were compelled to procure those not akin to take care of her. It was even said that the presence of her father in the room aggravated the violence of her paroxysms. But when out of the fits, all this aversion, all this morbid sensibility of telling colors by the touch, of hearing a whisper in another room, and of smelling less than a drop of essence of lemon through two beds on which she lay, vanished all at once, and she was pretty Nancy Hazard again, who finally got well and got married.

I have already remarked that the cases of Mrs. Cass and Jane C. Rider might be more marvellous than the one which I saw myself, and some further particulars in relation to their cases may here be appropriate.

Dr. Belden's patient, Jane C. Rider, was called the Springfield somnambulist, and was visited by clergymen and great numbers of people. When she came out of a fit, nothing done or said in it was at all recollected; but when another paroxysm came on, she remembered what was said and done in a preceding one. Dr. Belden says, "Her natural disposition was mild and amiable; but in her paroxysms she was commonly peevish and irritable." She would repeat poetry in her fits which she did not remember a word of when out of them; her sleeping memory being better than her waking one. She could see equally well in the dark as in the light, and generally supposed it was day; as a proof of which, when it was bed-time, and she was reminded that it was time for her to retire, her reply was, "What! go to bed in the day-time!" Attempts made to arouse her from her somnambulism were uniformly unsuccessful; she heard, felt and saw, or at any rate discerned, but awoke not. A pailful of cold water was once thrown on her, when she exclaimed, "*Why do you wish to drown me?*" went to her chamber and exchanged her dress, came down again, but awoke not. She had a pain in her head and an extremely tender spot upon it. Dr. B. took her to his own house, and there were usually from ten to twenty visitors in her room.

But her extreme and surprising faculty of discerning, so as to read with her eyes closed and in a dark room, claims our physiological notice, study and investigation; for, thus situated, she read a great number of cards, some of which were so obscurely written that the people with open eyes, and all alert, could scarcely make them out. "She told the date of coins, even when the figures were nearly obliterated," and threaded a needle for a lady who asked her, all with her eyes shut, and a handkerchief tied before them. And now, something like one hundred persons visited her in a day, and to "make the assurance doubly sure," another handkerchief was tied below the former, without any diminution of her describing what others could scarcely discern. Yet afterward, to further test her *sixth sense*, that of feeling without contact, and seeing when blinded, a large black silk handerchief was folded, and cotton batting so introduced as to be directly over her eyes, and to fill the cavity each side of her nose completely; various names were then written on cards, by most persons in the room, and presented to her, which she read as soon as presented. One gentleman present wrote his name in so small letters that no one could read them at the usual distance from the eye. "As soon as the paper was put into her hand she pronounced the name."

On one occasion, in a room made so dark that not a ray of light could penetrate, the fire was extinguished, and the lamps carried out. Two books were then presented to her, one of which it was known she had never seen before, yet "she immediately told the titles of both."

About this time she left Springfield, and was received into the Insane Hospital in Worcester, and we now come to what that acute and talented observer, Dr. Woodward, reports, viz.: Her pulse was 72 in a minute, soft and small; the extremities were cold, and at the commencement of the paroxysm the sleep was disturbed by sobbing and groaning; the breathing was interrupted and anxious, and she was uneasy and in perpetual motion. She told time by a watch with her eyes closed. Her eyes were then covered with a thick cotton handkerchief, folded so as to make eight or ten thicknesses, and the spaces below the bandage filled with black velvet. A small volume was then put into her hand: "Jane began at the paragraph indicated, and read distinctly, audibly, and correctly, not, however, without a slight degree of hesitation at the most difficult words, nearly the whole page." A game of back gammon was then proposed, which she said she knew nothing about, but consented to learn it, and with a little assistance soon acquired a knowledge of its principles; and in another paroxysm in the afternoon of the same day in which she played at first, she won the sixth game of Dr. Butler, who is an experienced player; and all this was done when her eyes were closed and the bandage and velvet before them. The doctor says, "When she threw the dice, she called the numbers distinctly, and immediately made the moves without any hesitation. But most surprisingly curious! when she came out of the fit, and was asked to play backgammon, she replied that she never saw it played in her life, and was entirely ignorant of the game; and on trial, it was found that she could not even set the men.

After her extraordinary acuteness of vision ceased, which it did while she was in the hospital, she continued to rehearse passages of poetry and sing songs and hymns, all which she was unable to do in her lucid intervals.

An emetic, given while in the hospital, appears to have been the most beneficial of any medicine she took, and Dr. Woodward was inclined to refer the disorder considerably to the stomach, as improper food appeared to sometimes bring on, and at other times to aggravate, her paroxysms. The brain was, however, seemingly implicated, as evinced by tenderness of the scalp, headache, and the amazing sensibility of her perceptions, and was considered the proximate seat of difficulty, which exciting causes were prone to develope.

These cases, with that of Rachel Baker, and one which I have since noticed from the pen of A. B. Shipman, of Syracuse, N. Y., seem to prove that all the laws of mentality have not as yet been grasped; that the mind, in fact, has a duality of powers independent of each other.

Since the finest injections cannot be forced from the arteries into the veins, we must view anatomy and physiology as not fully developed by our great masters. The anatomical fact seems also to be in abeyance, why, in the dead subject, all the blood is found in the veins, and the arteries empty; which would seem to show that dying, in every form, is by bleeding to death—venous blood being as incapable of containing life in the veins, as if spilt on the ground. Thus the doors, both mental and anatomical, seem yet wide open to the physiologist.

I would in this place notice, relative to the strange-colored matter which was discharged from Nancy Hazard's hand, that a very respectable physician, resident in the same county in which she lived, informed me that he had a case of a man bitten in the heel by a spider, that an abscess formed in consequence, which he lanced, and that the matter discharged was green. A newspaper, if I mistake not, gave a similar account of a case which occurred in Georgia, some years past. And a case of death from the bite of a spider is found detailed in the Boston Medical and Surgical Journal of October 23d, 1844.

Somnambulism differs from sleep in the faculty of hearing, conversing, acting systematically, and traveling from place to place. Dreams in common sleep are remembered, but what happens in somnambulism is not.

The case of Rachel Baker occurred eleven years after the publication of Nancy Hazard's case. The late Samuel L. Mitchell, M. D., of New York, has given some interesting particulars respecting the preaching, exhorting and praying, in her fits of reverie or somnambulism, not a word of which she remembered when awake. It was remarked of Rachel, that she appeared to have two souls, her waking soul not remembering a single thing that her sleeping soul did or said, and *vice versa*.

In the case of Mrs. Cass, I omitted to say, that she at one time was entirely blind, which goes to prove that the visual rays which usually go from the brain to the eye had a transfer or metastasis to her other nerves; for when thus blind, and with her face turned away, she could still tell every person who came into her room, and any and every trivial thing that took place. She could also walk her room without coming in contact with the furniture or anything in her way. Her case is given at length by two respectable physicians, Drs. Bernard and Colby, the latter of whom mentions that this singular endowment, which I have called the

*sixth sense*, is mentioned by Dr. Good. One of her physicians also says, that, "guided by her internal sensation, she directed means for her recovery." These means were cupping four times on her stomach, and the use of the warm bath, by which she ultimately recovered. Her sufferings were great, and one feature of them is worthy of notice; viz., that when entirely blind with her eyes, she was still so distressed at the approach of light, that her attendants were obliged to keep her room in darkness. This is another proof of the metastasis of the eyesight to other sets of nerves.

I shall conclude cases of this kind by a reference to one which exceeds them all, and is related by a clergyman (the Rev. Mr. Glover, of Liverpool), from his own examination.

Miss M'Evoy, the patient, became blind in the month of June, 1816. The cause of her blindness is imputed to dropsy of the brain, of which she was relieved, we are told, by the discharge of water from the ears and nostrils, which did not, however, relieve her of blindness. The October following she accidentally discovered that she could read by touching the page of a book printed by unraised letters; and when the Rev. Mr. G. visited her, he found this to be the case, and she thus read fine print to him; her age was about 17.

But he did not fail of testing her surprising powers in several modes; and to prove that her eyes had nothing to do in the matter, he had her blindfolded, and now he put her *finger-sight* to several tests. He first enclosed six wafers between two plates of common window-glass, and he tells us that when thus blind and blindfolded, on touching the glass, "she accurately told the color of each." She told him the time of day by feeling the crystal of a watch, and would describe passengers and things in the street by touching the panes of the window.

I thought, as did every one who heard of it, that the accident in blasting rocks, by which a young man at Cavendish, Vermont, had an iron bolt, three feet long and nearly an inch in diameter, driven quite through his brain, and who lived, and afterward visited Boston, was, and would forever remain, quite unparalleled. But I have since seen one given by Dr. Macartney, quite its equal, which I shall give in his own words:—

"He had known," he said, "an instance where a pitchfork had been driven into the eye of a man, had pierced the brain, and fixed itself so firm at the top of his head, that it was obliged to be hammered out from the opposite bone; and the man's mental functions never were disturbed by it, and he recovered and lived for some time."—*Boston Medical and Surgical Journal.*

*Lebanon, Conn., April*, 1857.

